# The Pore-Forming Subunit C2IIa of the Binary *Clostridium botulinum* C2 Toxin Reduces the Chemotactic Translocation of Human Polymorphonuclear Leukocytes

**DOI:** 10.3389/fphar.2022.810611

**Published:** 2022-02-11

**Authors:** Julia Eisele, Simone Schreiner, Joscha Borho, Stephan Fischer, Sebastian Heber, Sascha Endres, Maximilian Fellermann, Lisa Wohlgemuth, Markus Huber-Lang, Giorgio Fois, Michael Fauler, Manfred Frick, Holger Barth

**Affiliations:** ^1^ Institute of Pharmacology and Toxicology, Ulm University Medical Center, Ulm, Germany; ^2^ Institute of General Physiology, Ulm University, Ulm, Germany; ^3^ Institute of Clinical and Experimental Trauma Immunology, Ulm University Medical Center, Ulm, Germany

**Keywords:** binary C2 toxin, *Clostridium botulinum*, transport subunit C2IIa, trans-membrane channel, polymorphonuclear leukocytes, chemotaxis, migration

## Abstract

The binary C2 toxin of *Clostridium* (*C*.) *botulinum* consists of two non-linked proteins, the enzyme subunit C2I and the separate binding/transport subunit C2II. To exhibit toxic effects on mammalian cells, proteolytically activated C2II (C2IIa) forms barrel-shaped heptamers that bind to carbohydrate receptors which are present on all mammalian cell types. C2I binds to C2IIa and the toxin complexes are internalized via receptor-mediated endocytosis. In acidified endosomal vesicles, C2IIa heptamers change their conformation and insert as pores into endosomal membranes. These pores serve as translocation-channels for the subsequent transport of C2I from the endosomal lumen into the cytosol. There, C2I mono-ADP-ribosylates G-actin, which results in depolymerization of F-actin and cell rounding. Noteworthy, so far morphological changes in cells were only observed after incubation with the complete C2 toxin, i.e., C2IIa plus C2I, but not with the single subunits. Unexpectedly, we observed that the non-catalytic transport subunit C2IIa (but not C2II) alone induced morphological changes and actin alterations in primary human polymorphonuclear leukocytes (PMNs, *alias* neutrophils) from healthy donors *ex vivo*, but not macrophages, epithelial and endothelial cells, as detected by phase contrast microscopy and fluorescent microscopy of the actin cytoskeleton. This suggests a PMN selective mode of action for C2IIa. The cytotoxicity of C2IIa on PMNs was prevented by C2IIa pore blockers and treatment with C2IIa (but not C2II) rapidly induced Ca^2+^ influx in PMNs, suggesting that pore-formation by C2IIa in cell membranes of PMNs is crucial for this effect. In addition, incubation of primary human PMNs with C2IIa decreased their chemotaxis *ex vivo* through porous culture inserts and in co-culture with human endothelial cells which is closer to the physiological extravasation process. In conclusion, the results suggest that C2IIa is a PMN-selective inhibitor of chemotaxis. This provides new knowledge for a pathophysiological role of C2 toxin as a modulator of innate immune cells and makes C2IIa an attractive candidate for the development of novel pharmacological strategies to selectively down-modulate the excessive and detrimental PMN recruitment into organs after traumatic injuries.

## Introduction

The C2 toxin of *Clostridium* (*C*.) *botulinum* is the prototype of the family of binary actin ADP-ribosylating toxins (Ohishi, 1983b; Aktories et al., 1986; Stiles et al., 2014). C2 toxin consists of two separate, non-linked proteins, which are secreted by the bacteria and assemble in solution or on the surface of eukaryotic target cells to form biologically active toxin complexes (Ohishi, 1983a; Ohishi et al., 1984; Ohishi and Miyake, 1985; Kurazono et al., 1987; Ohishi and Yanagimoto, 1992; Barth et al., 2000). One toxin subunit is the enzymatically active protein C2I (∼50 kDa) (Aktories et al., 1986; Fuji et al., 1996; Barth et al., 1998a; Barth et al., 1998b), the other one is the binding/transport subunit C2II (∼80 or ∼100 kDa, depending on the *C. botulinum* strain) (Ohishi, 1987; Barth et al., 2000; Sterthoff et al., 2010). It was observed earlier by various groups that only the combination of C2I and C2II, but not the single subunits alone had cytotoxic effects when applied to cultured cells (Ohishi, 1983a; Ohishi et al., 1984; Barth et al., 1998a; Barth et al., 1998b; Barth et al., 2000) and the combination of C2I and C2IIa induced delayed caspase-dependent cell death in dividing cultured cells after 24–48 h (Heine et al., 2008).

We and others investigated the role of C2II in detail and found that the protein consists of four functionally different domains (Schleberger et al., 2006): The N-terminal domain 1 contains a cleavage site for proteases (Ohishi, 1987; Barth et al., 2000), domain 2 serves as binding site for C2I (Kaiser et al., 2006; Schleberger et al., 2006; Lang et al., 2008), domain 3 is crucial for membrane insertion (Blöcker et al., 2003; Knapp et al., 2016) and the C-terminally located domain 4 binds to the receptor on the surface of target cells (Blöcker et al., 2000). Limited proteolysis by host proteases removes a ∼20 kDa peptide from the N-terminal domain of C2II, which generates the biologically active species C2IIa (Ohishi, 1987; Barth et al., 2000). C2IIa, but not C2II, forms barrel-shaped heptamers (Barth et al., 2000), which bind to complex and hybrid carbohydrate receptors on cells, which are present on all mammalian cell types (Eckhardt et al., 2000). Therefore, all cell types bind C2IIa and are sensitive towards C2 toxin. Moreover, C2IIa but not C2II forms pores in lipid bilayer membranes *in vitro* (Schmid et al., 1994). C2I binds to C2IIa but not to C2II (Barth et al., 2000; Kaiser et al., 2006), and subsequently the C2I/C2IIa complexes are internalized into cells via receptor-mediated endocytosis (Barth et al., 2000; Nagahama et al., 2009; Pust et al., 2010; Takehara et al., 2017). Noteworthy, C2IIa alone is also internalized by endocytosis (Nagahama et al., 2017). Following endocytosis of the C2IIa/C2I complexes, C2IIa mediates the release of C2I from early acidic endosomes into the cytosol (Barth et al., 2000; Barth et al., 2002). According to the current model, driven by the acidic conditions in the endosomal lumen, C2IIa heptamers change their conformation, insert into the endosomal membranes and form trans-membrane pores, which serve as translocation-channels for the transport of C2I across the endosomal membrane (Barth et al., 2000; Bachmeyer et al., 2001; Blöcker et al., 2003a; Blöcker et al., 2003b). In the cytosol, C2I mono-ADP-ribosylates G-actin at position arginine-177 (Aktories et al., 1986; Vandekerckhove et al., 1988), which turns G-actin into a capping protein that prevents further addition of G-actin to the growing end of actin filaments (Aktories et al., 1989; Uematsu et al., 2007). This results in depolymerization of F-actin and cell-rounding, which leads to a breakdown of biological barriers *in vivo* (Ohishi, 1983a; Ohishi, 1983b; Kurazono et al., 1987).

Recently, the initial steps of the internalization of C2 toxin were investigated in more detail by Nagahama and co-workers. According to their model built on observations in MDCK cells, binding of C2IIa to cells triggers a series of events resulting in the generation of microdomains in the plasma membrane, which facilitate the uptake of C2 toxin. Taken together, after binding to cells, a portion of cell-bound C2IIa forms pores in the plasma membrane, which causes Ca^2+^ influx into the cells (Nagahama et al., 2021). This Ca^2+^ influx triggers lysosomal exocytosis of enzymes including the cysteine protease cathepsin B (Nagahama et al., 2021). Cathepsin B activates acid sphingomyelinase, which hydrolyzes sphingomyelin and converts it to ceramide, thereby generating ceramide-rich microdomains in the plasma membranes that bud into cells, generate endosomal vesicles and thereby facilitate C2 toxin endocytosis (Nagahama et al., 2017). Moreover, this group reported that cells were protected from C2 toxin in the presence of inhibitors against acid sphingomyelinase and in the absence of calcium (Nagahama et al., 2017). Treatment of cells with heat-inactivated C2IIa or the neutralization of C2IIa with a specific antibody did not result in the production of ceramide (Nagahama et al., 2017). Thus, the authors concluded that C2IIa is essential to induce Ca^2+^ influx, lysosomal exocytosis, and finally endocytosis of C2 toxin, which extends the functions of C2IIa.

However, in previous studies, no morphological changes or cytotoxic effects were observed when cells were incubated with C2IIa in the absence of C2I (Ohishi, 1983a; Ohishi et al., 1984; Barth et al., 1998a; Barth et al., 1998b; Barth et al., 2000; Heine et al., 2008), suggesting that the pore formation in the plasma membrane does not result in cytotoxicity and might be limited to trigger toxin uptake. In the present study, we investigated the effect of C2IIa on cells in more detail using primary human PMNs. It was reported earlier that complete C2 toxin (C2IIa + C2I) inhibits migration of activated human neutrophils *ex vivo* (Norgauer et al., 1988) and that treatment of human PMNs with C2IIa + C2I, but not with C2IIa or C2I alone rapidly inhibited signal transduction via the N-formyl peptide receptor (Norgauer et al., 1989; Grimminger et al., 1991). Our results revealed that treatment of these cells with C2IIa alone, but not with the biologically inactive precursor C2II, caused obvious morphological changes and alterations in the actin cytoskeleton, which were prevented by C2IIa pore blockers. Moreover, incubation of primary human PMNs with C2IIa induced Ca^2+^ influx into the cytosol and decreased their chemotaxis through porous cell-culture inserts in the absence and presence of barrier-forming, co-cultured human endothelial cells *ex vivo*. In line with earlier studies, there were no detectable effects of C2IIa on the morphology or viability of human endothelial and epithelial cells or macrophages.

## Materials and Methods

### Purification of C2I and C2II and Activation of C2IIa

*E. coli* BL21 were transformed with pTRC99A:His_C2II, in which the amino acids ENLYFQ have been inserted C-terminally after Lys-181 to increase the efficiency of activation to C2IIa at 4°C. Five millilitre of LB-medium (1% tryptone, 0.5% yeast extract, 1% NaCl and 100 μg/ml ampicillin) were inoculated with a single colony and incubated at 37°C and 180 rpm for 5 h. A 150 ml LB-medium over-night culture was inoculated with the 5 ml preculture and incubated under the same conditions. 4 × 1l of main culture were each inoculated with 30 ml of the overnight culture and grown to an OD_600_ of 0.6 under the same conditions. Expression of His_C2II was induced by addition of 0.5 mM Isopropyl β-d-1-thiogalactopyranoside and performed at 16°C and 180 rpm overnight. The bacteria were harvested by centrifugation at 5,500 rcf and 4°C for 10 min and resuspended in 40 ml of NPI-20 (50 mM NaH_2_PO_4_, 300 mM NaCl, 20 mM imidazole, pH 8.0). 1% phenylmethylsulfonyl fluoride (100 mM in Ethanol) was added for protease inhibition. Bacteria were lysed by sonication (10 × 20 s pulses with 30 s breaks). The lysate was centrifuged at 13,000 rcf and 4°C for 20 min. The supernatant containing the protein of interest was filtered through 0.2 µm syringe filters and incubated overnight at 4°C with 1 ml bed-volume of PureCube 100 INDIGO Ni-agarose (Cube Biotech, Monheim am Rhein, Germany). Midi Plus columns (Cube Biotech, Monheim am Rhein, Germany) were used for protein extraction from Ni-agarose according to the instructions of the manufacturer at 4°C. NPI-20 was used as washing- and equilibration-buffer, NPI-250 (50 mM NaH_2_PO_4_, 300 mM NaCl, 250 mM imidazole, pH 8.0) was used as elution buffer. Fractions were analyzed by SDS-PAGE with subsequent Coomassie-staining. The fractions with high His_C2II concentration and low levels of impurities were combined. The protein was rebuffered in PBS (137 mM NaCl, 2.7 mM KCl, 8 mM Na_2_HPO_4_, 1.8 mM KH_2_PO_4_, pH 7.4) to a dilution factor <1:1,000 and concentrated by centrifugation (Vivaspin 20, 30.000 MWCO; Sartorius, Göttingen, Germany). The protein was frozen in liquid N_2_ and stored at −80°C until activation. For proteolytic activation, the protein was thawed on ice. Trypsin was added to a stochastic ratio of 2:3 (n(trypsin):n(His_C2II)) and incubated for 15 min at 4°C for activation of His_C2II to C2IIa. Trypsin activity was inhibited by addition of an equal amount of trypsin inhibitor (m(trypsin) = m(trypsin inhibitor)) and incubation on ice for 1 h. Aggregated C2IIa was removed by centrifugation at 21,000 rcf for 10 min at 4°C. C2IIa purity and concentration was analyzed by SDS-PAGE with subsequent Coomassie staining and densitometric analysis. C2I was purified as described earlier (Barth et al., 1998b). The purity and identity of C2I, C2II and C2IIa were confirmed by SDS-PAGE and Western blotting with specific antibodies against C2I and C2II as described before (Barth et al., 1998b; Barth et al., 2000).

### Cell Culture

Human umbilical vein endothelial cells (HUVEC) were obtained from ATCC^®^ (Manassas, Virginia, United States) and maintained in Endothelial Cell Growth Medium MV2 containing 10% Growth Medium MV2 Supplement Mix (both PromoCell, Heidelberg, Germany) and 1% penicillin/streptomycin (Thermo Fisher Scientific, Ulm, Germany) at 37°C and 5% CO_2_. For chemotaxis assays, HUVECs were seeded on 0.3 cm^2^ translucent cell-culture inserts with a pore size of 3 µm (Sarstedt, Nümbrecht, Germany) coated with huAEC Coating Solution (InSCREENeX, Braunschweig, Germany) at a density of 5 × 10^4^ cells/insert. Chemotaxis assays were performed between 48 and 72 h after seeding, when transendothelial electrical resistances of at least 15 Ω cm^2^ (cellZscope, nanoAnalytics, zMünster, Germany) were reached. HUVEC were used in experiments until passage 9.

HeLa cells were obtained from DSMZ (Braunschweig, Germany) and maintained in Minimal Essential Medium (MEM) supplemented with 10% fetal calf serum (FCS), 1% Penicillin-Streptomycin, 1 mM Sodium Pyruvate, 0.1 mM MEM NEAA (all Thermo Fisher Scientific, Ulm, Germany) and 2 mM L-glutamine (Biochrom GmbH, Berlin, Germany) at 37°C and 5% CO_2_. For cell seeding, see section *Cytotoxicity and Cell Viability Assays*.

Murine macrophage-like J774A.1 cells were obtained from DSMZ (Braunschweig, Germany) and maintained in Dulbecco’s Modified Eagle’s Medium (DMEM) containing 10% FCS, 1% penicillin/streptomycin, 0.1 mM MEM NEAA (all from Thermo Fisher Scientific, Ulm, Germany) and 2 mM L-glutamine (Biochrom GmbH, Berlin, Germany) at 37°C and 5% CO_2_. For cell seeding, see section *Cytotoxicity and Cell Viability Assays*.

### Isolation of Human Polymorphonuclear Cells (PMN)

PMNs were isolated from blood of healthy volunteers (ethics votum 24/16 of the Local Independent Ethics Committee of the University of Ulm). PMNs were purified using OptiPrep™ (Alere Technologies AS, Oslo, Norway) density gradient-medium following modified methods of the application sheet C12 (OptiPrep™ Application Sheet C12; 8th edition, January 2020) under endotoxin-free conditions. Isolation was performed at room temperature. One millilitre of 6% high-molecular weight dextran solution (Sigma-Aldrich, Hamburg, Germany) was added to 9 ml of freshly drawn blood, collected using S-Monovette^®^ 7.5 ml K3 EDTA and Safety-Multifly®-Needle (both Sarstedt, Nümbrecht, Germany) and mixed by several very gentle inversions. After 40 min, the supernatant (leukocyte-rich plasma) was aspirated and layered over a density gradient. For this purpose, two centrifugation media with densities of 1.077 g/ml and 1.090 g/ml were prepared by mixing OptiPrep™-medium with appropriate amounts of BSS-A (balanced salt solution A: 136.21 mM NaCl, 5.55 mM (D+)-glucose, 2.7 mM phosphate buffer, 10.65 mM HEPES, 5.4 mM KCl, pH 7.4 at room temperature, 285–295 mosmol/kg). After centrifugation (800 rcf, 25 min), polymorphonuclear leukocytes (PMN) were harvested from the lower interface of the gradient layer and gently diluted with an equal volume of BSS, followed by centrifugation for 10 min at 300 rcf. The pellet was resuspended in 5 ml RPMI Medium 1640 HEPES (IrvineScientific, Santa Ana, United States) with 10% FBS (Gibco by Thermo Fisher Scientific, Waltham, Massachusetts, United States). Cells were stained with calcein AM (Thermo Fisher Scientific, Ulm, Germany) at a final concentration of 200 nM for 20 min in the dark. Counting was performed using Countess™ II FL (Thermo Fisher Scientific, Ulm, Germany). PMN cell suspensions were adjusted to a density of 4 × 10^4^ cells/µl with BSS-B (Balanced Salt Solution B: 132.79 mM NaCl, 5.55 mM (D+)-Glucose, 2.6 mM Phosphate buffer, 10.3 mM HEPES, 5.2 mM KCl, 1.9 mM CaCl_2_, 1.3 mM MgSO_4._, pH 7.0 at 37°C, 290 mosmol/kg). PMN isolations had a purity above 88% (mean 94.0% ± SD 4.4%) as confirmed by cytospin preparations (THARMAC^®^ Laboratory Solutions, Wiesbaden, Germany) and Pappenheim-staining using the Hemacolor^®^ staining kit (Merck, Darmstadt, Germany) according to the manufacturer’s instructions.

### Cytotoxicity and Cell Viability Assay

For morphology- and viability-based assays, cells were seeded (100 µl/well; HeLa: 4.25 × 10^4^ cells/ml, HUVEC: 7.5 × 10^4^ cells/ml, J774A.1: 7.7 × 10^4^ cells/ml, PMNs: 5 × 10^5^ cells/ml) in 96 well plates. HeLa and J774A.1 cells were grown for 1 day, HUVECs for 3 days, and PMNs (in RPMI Medium 1640 HEPES with 10% FBS) were seeded directly prior to the experiment. For intoxication, the medium was exchanged with fresh medium (100 µl) containing the respective toxin components at indicated concentrations and the cells were incubated at 37°C and 5% CO_2_ for indicated time periods. To analyze morphological changes in cells, images were acquired on a LEICA DMi1 microscope (Leica Microsystems CMS GmbH, Wetzlar, Germany) connected to a LEICA MC170 HD camera (Leica Microsystems Ltd, Heerbrugg, Switzerland). Morphologically changed cells (defined as those cells that look substantially different from the majority of cells in the untreated control) were counted using neuralab software (Neuralab.de) and their percentage on the total number of cells per picture (one picture per well, three wells per condition) was calculated. Cell viability was determined by using the Cell Titer 96^®^ AQueous One Solution Cell Proliferation Assay (MTS) from Promega (Walldorf, Germany), according to the manufacturer’s instructions. Mock- and DMSO [20% (v/v)]-treated cells served as negative and positive control for cell viability, respectively. Following an incubation period of 24 h at 37°C, MTS [3-(4,5-dimethylthiazol-2-yl)-5-(3-carboxymethoxyphenyl)-2-(4-sulfophenyl)-2H-tetrazolium] was added to the medium of each well and the absorption was measured after 1 h at 490 nm using a microtiter plate reader.

### Flow Cytometry Analysis of PMNs

Primary human PMNs were resuspended in phosphate-buffered saline containing calcium and magnesium (PBS, Gibco Thermo Fisher, Darmstadt, Germany) that was titrated to a physiological pH of 7.3. The cell concentration was adjusted to 2 × 10^6^ cells/ml. PMNs were either incubated with phosphate buffer (PBS, for control), with N-formyl-Met-Leu-Phe (fMLF, 1 µM), C2I (1 μg/ml), C2IIa (2 μg/ml), or the combination of fMLF (1 µM) with either C2I (1 μg/ml) or C2IIa (1 μg/ml). PMNs were incubated at 37°C and measured after 5, 30, and 60 min using a Canto II flow cytometer (BD Biosciences, Heidelberg, Germany) as previously described (Hug et al., 2021). Doublets were excluded based on the linearity of the forward scatter area (FSC-A) and height. PMNs consisting mainly of neutrophils (at least 5 × 10^3^ cells/measurement) were identified based on their FSC and side scatter properties.

### Analysis of Cytosolic Ca^2+^ by Flow Cytometry

Cells were suspended at a density of 1 × 10^7^ cells/ml in FACS buffer (143 mM NaCl, 6 mM KCl, 1 mM MgSO_4_, 20 mM HEPES/NaOH, pH 7.4, 1 mM CaCl_2_, and 5.6 mM glucose) and incubated for 45 min at 37°C and 10% CO_2_ with 5 μM Indo-1-AM (I1223, Thermo Fisher Scientific, Waltham, Massachusetts, United States). The Ca^2+^ measurements were performed on a BD FACS Celesta™. In brief, 1 × 10^6^ cells/sample in 0.5 ml of FACS buffer were pre-warmed for 10 min at 37°C. Next, the baseline intracellular cytosolic Ca^2+^ concentration ([Ca^2+^]_i_) was measured for 2 min before addition of C2IIa (5 μg/ml), C2II (5 μg/ml) or C2I (1 μg/ml). After an additional 4 min, 2 μM ionomycin (I0634, Sigma-Aldrich, Hamburg, Germany), a Ca^2+^-selective ionophore (Beeler et al., 1979), was added to the samples to obtain maximum [Ca^2+^]_i_. Cells were kept at 37°C prior and during their injection from the sample tube into the flow cytometer instrument. The fluorescence ratio of Indo-1 violet (emission wavelength about 400 nm, Ca^2+^-bound) to Indo-1 blue (emission wavelength about 475 nm; Ca^2+^-free) following excitation at about 355 nm was measured by using FACSDiva™ software (BD Biosciences). For data evaluation, the experimental results contained in FCS files generated by the FACSDiva™ software (BD Biosciences) were imported into FlowJo™ v10.8 Software (BD Life Sciences) and analyzed using FlowJo™ kinetics platform. The individual points of the curves shown in Figure 3A correspond to the times in full seconds *versus* the medians of all fluorescence ratios representing [Ca^2+^]_i_, following normalization according to the maximal values reached after addition of 2 μM ionomycin.

### Chemotaxis-Transmigration Assays With Human PMNs

0.3 cm^2^ translucent cell-culture inserts with a pore size of 3 µm were coated with human fibronectin (1% solution in PBS, Advanced BioMatrix, Carlsbad, United States). 2 × 10^5^ calcein AM stained PMNs were seeded in BSS-B into cell culture inserts (pore size 3 µm, Sarstedt, Nümbrecht, Germany) placed in 24 well plates (Greiner Bio-One, Kremsmünster, Austria) containing BSS-B. Cells were incubated for 2 h at 37°C in the presence or absence of C2IIa (2 μg/ml), C2I (1 μg/ml), the binary toxin (2 μg/ml C2IIa + 1 μg/ml C2I) or cytochalasin B (5 μg/ml) (Sigma-Aldrich, Hamburg, Germany), serving as a positive migration-inhibitory control.

For transmigration assays through endothelial cell layers, control or toxin-treated PMNs were seeded on HUVEC monolayers with transendothelial resistances of at least 15 Ω cm^2^ (see section *Cell Culture*) subsequently to pre-incubation of HUVECs with TNFα (10 ng/ml, Biomol, Hamburg, Germany). All measurements were performed in BSS-B.

Calcein fluorescence was repetitively measured from the top using a Tecan Infinite^®^ M200 plate reader (Tecan Group, Männedorf, Switzerland) at 37°C (excitation 470–475 nm, emission measured at 513 nm after a lag time of 2 µs). fMLF (10 nM, Sigma-Aldrich, Hamburg, Germany) was applied to the lower chamber to trigger chemotaxis. Final volumes were 100 µl or 500 µl in the upper or lower compartments, respectively.

The chemoattractant fMLF triggered chemotaxis from the upper to the lower compartment of cell culture inserts. Therefore, the upper calcein-fluorescence signal declined with the transmigration of calcein-stained PMN through the membrane. Fluorescence data were normalized to the initial fluorescence intensity. Statistical analysis was performed for steady-state values after 90–95 min.

### Measurement of the Effect of Binary C2 Toxin and Its Single Components on PMN Membrane Integrity

Isolated calcein-stained PMNs were seeded on a µ-slide 8 well slide (Ibidi) at a concentration of 1 × 10^5^ cells/well. Cells were incubated for 1 h in bath solution (140 mM NaCl, 5 mM KCl, 1 mM MgCl_2_, 2 mM CaCl_2_, 5 mM glucose, 10 mM Hepes, pH 7.4) at 37°C 5% CO_2_ to allow them to settle at the bottom of the well. Live cell fluorescence imaging was performed on an iMic digital microscope (Till Photonics, Germany) using a 488 nm excitation filter. Images were acquired with a 20x uPlanSapo objective (Olympus, Japan) at a rate of 0.3 Hz using iMic Online Analysis software (Till Photonics) for 30 min after stimulation with C2IIa (2 μg/ml), C2I (1 μg/ml), the binary C2 toxin (2 μg/ml C2IIa + 1 μg/ml C2I), ionomycin (2 µM, Sigma Germany) or saponin [0.2% (w/v)]. Images were captured for 30 min after stimulation at a rate of 0.3 Hz. Acquired images were analysed with FIJI software (Schindelin et al., 2012). Briefly, image stacks were aligned to compensate movement, subsequently images were background subtracted and a region of interest (ROI) was drawn around each cell and fluorescence was measured for each ROI in all images of the stack. Cells moving out of their ROI were excluded from the analysis. Further analysis was performed in Excel (Microsoft Office 365). Data was normalized to the average fluorescence intensity before treatment. Values before treatment (F0) and after 30 min (F30) were compared.

### Assessment of Barrier-Integrity of Endothelial Cell Monolayers

In some transmigration experiments through HUVEC cell monolayers, fluorescence-labelled dextrans were added to the upper compartment. A damage of the endothelial cell layer, e.g., eventually caused by PMN transmigration, leads to an increased diffusion of dextrans to the lower compartment. Alexa Fluor™ 647-labelled 10 kDa and Texas Red™-labelled 70 kDa dextrans (both Thermo Fisher Scientific, Ulm, Germany) were added at final concentrations of 5 and 25 μg/ml, respectively. Fluorescence signals were measured with the top sensor of the Tecan Infinite M200 in addition to the calcein signal from PMN transmigration (see section *Chemotaxis-Transmigration Assay*). Raw fluorescence data was normalized to respective initial values. A stronger decline of normalized fluorescence intensity was interpreted as increased diffusion.

### Statistics

Data was analyzed with Microsoft Excel (Microsoft Office 365) and GraphPad Prism 7 and 9 (GraphPad Software, San Diego, California, United States). Statistical significance was tested by applying the Kruskal–Wallis test with Dunn’s correction for multiple comparison if not otherwise indicated in figure legends. *p*-values below 0.05 were considered statistically significant.

## Results

### Treatment With C2IIa Results in a Changed Morphology of Primary Human PMNs

**FIGURE 1:**
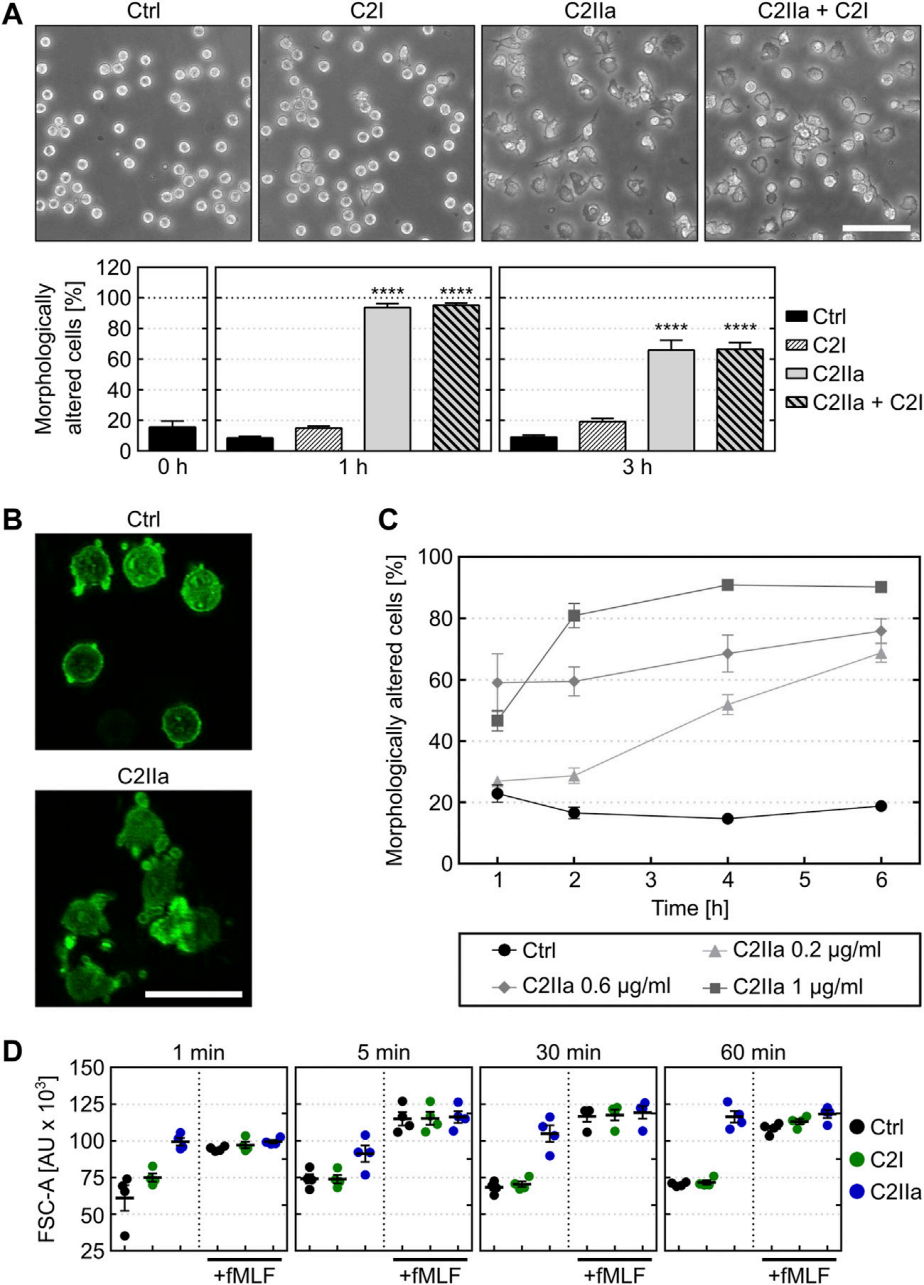
Effect of C2IIa on the morphology of primary human neutrophils (PMNs) ex vivo. **(A)** Upper panel: Representative pictures show the morphology of PMNs incubated for 3 h at 37°C with C2IIa (2 μg/ml), C2I (1 μg/ml), both components (2 μg/ml C2IIa + 1 μg/ml C2I) or without any protein as control (Ctrl). Scale bar: 100 µm. Lower panel: Quantitative evaluation of morphologically altered PMNs after 1 and 3 h treatment at 37°C with C2IIa (2 μg/ml), C2I (1 μg/ml), both components (2 μg/ml C2IIa + 1 μg/ml C2I) or without any protein as control (Ctrl). Data show the results of 5 independent experiments, each with 3 technical replicates. Values represent the mean ± SEM (n = 15). Significance was tested against the untreated control (Ctrl) at each individual respective time point using Kruskal–Wallis test with Dunn’s correction for multiple comparison (****p < 0.0001). Untreated cells (Ctrl) did not change their morphology over the time course of the experiment. At time point 0, all cells showed the same morphology as the untreated control cells when the respective proteins were applied to the individual groups of the cells. **(B)** Representative pictures of actin-stained (phalloidin-FITC) PMNs after 3 h incubation at 37°C with 1 μg/ml C2IIa or without any protein (Ctrl). Scale bar: 25 µm. **(C)** Quantitative analysis of morphological changes over time of PMNs treated with different concentrations of C2IIa or no C2IIa (Ctrl). Data show the results of 3 independent experiments, each with 3 technical replicates. Values represent the mean ± SEM (n = 9). **(D)** Flow cytometryc analysis of PMNs after treatment with C2IIa, C2I or PBS for control (Ctrl). Change of FSC was measured as surrogate for PMN cell size after incubation with C2I (1 μg/ml) or C2IIa (2 μg/ml). In parallel, PMNs were incubated with C2I (1 μg/ml) or C2IIa (2 μg/ml) in the presence of fMLF (1 µM), a chemoattractant peptide. PMNs from the same tube were analyzed after 1, 5, 30, and 60 min. Data is given as mean of four independent experiments ± SEM (n = 4).

**FIGURE 2:**
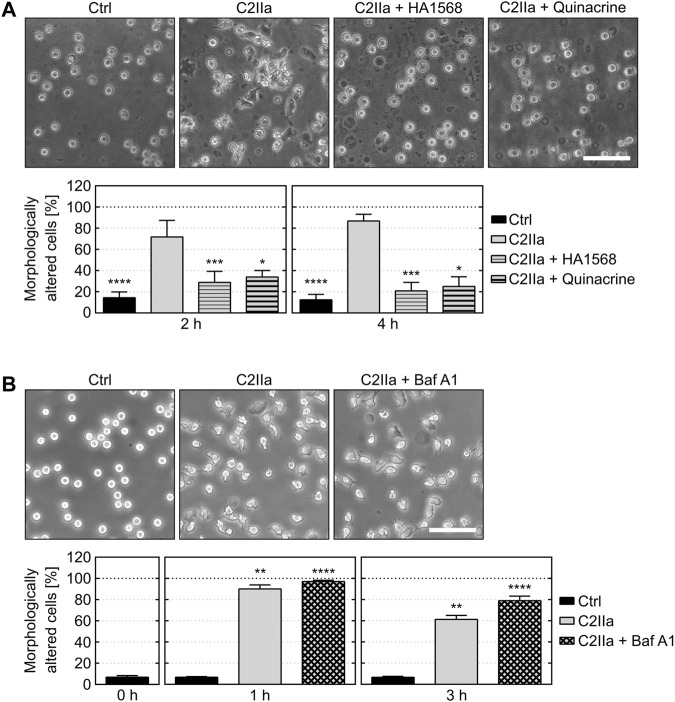
Effect of pore blockers and bafilomycin A1 on C2IIa-induced changes in PMN morphology. **(A)** The pore blockers HA1568 and quinacrine protect PMNs from C2IIa-induced morphological changes. Upper panel: Representative pictures show the morphology of PMNs after 4 h treatment (37°C) with C2IIa (1 μg/ml) in the presence or absence of the established pore blockers HA1568 (100 µM) and quinacrine (5 µM). Additional PMNs were left untreated as control (Ctrl). Scale bar: 100 µm. Lower panel: The percentage of morphologically changed PMNs was quantified after 2 and 4 h and is depicted as mean ± SEM (n = 9–15, i.e., 3–5 independent experiments with 3 technical replicates for each condition, respectively). Significance was tested against the sample treated with C2IIa only using Kruskal–Wallis test with Dunn’s correction for multiple comparison (*p < 0.05, ***p < 0.001, ****p < 0.0001). **(B)** Inhibition of endosomal acidification by bafilomycin A1 (Baf A1) has no impact on the C2IIa-induced morphological changes of PMNs. Upper panel: Representative pictures show the morphology of PMNs after 1 h treatment (37°C) with C2IIa (2 μg/ml) with or without Baf A1 (100 nM). Further PMNs were left untreated as control (Ctrl). Scale bar: 100 µm. Lower panel: The percentage of morphologically altered PMNs was quantified after the indicated time points and is depicted as mean ± SEM of three independent experiments, each with 3 technical replicates (n = 9). Significance was tested against the untreated control (Ctrl) using Kruskal–Wallis test combined with Dunn’s multiple comparison test (**p < 0.01, ****p < 0.0001).

The identity of the recombinant proteins C2I, C2II and C2IIa that were prepared for the experiments was confirmed by SDS-PAGE and Western blotting (Supplementary Figure S1). To analyze whether treatment with C2IIa has an effect on PMNs, cells were prepared from blood of healthy donors and incubated for 3 h in the presence or absence of C2IIa. In parallel, cells were incubated with C2IIa + C2I to confirm that these cells are sensitive for C2 toxin and able to bind and internalize C2IIa. As further control, cells were incubated with C2I alone. The morphology of the cells was documented by phase contrast microscopy and the percentage of morphologically altered cells quantified from the pictures. As shown in Figure 1A, cells treated with complete C2 toxin showed an altered morphology, which was in line with earlier observations (Barth et al., 1998a; Barth et al., 1998b; Barth et al., 2000; Heine et al., 2008). As expected, cells treated with C2I alone did not change their morphology compared to untreated control cells. However, the cells treated with C2IIa alone showed an altered morphology, which was also observed by fluorescence microscopy of the actin cytoskeleton (Figure 1B) and this effect was time- and concentration-dependent (Figure 1C, Supplementary Figure S2). Untreated cells did not change their morphology over time and remained on a basal level (see Figures 1A, 2B, Supplementary Figure S2). Additionally, the effect of C2IIa on human PMNs was analyzed by flow cytometry (Figure 1D). C2IIa but not C2I rapidly changed the size/morphology of PMNs. Moreover, C2IIa had no significant additional effect on the cellular size of PMNs that were at the same time stimulated with fMLF and C2IIa.

**FIGURE 3:**
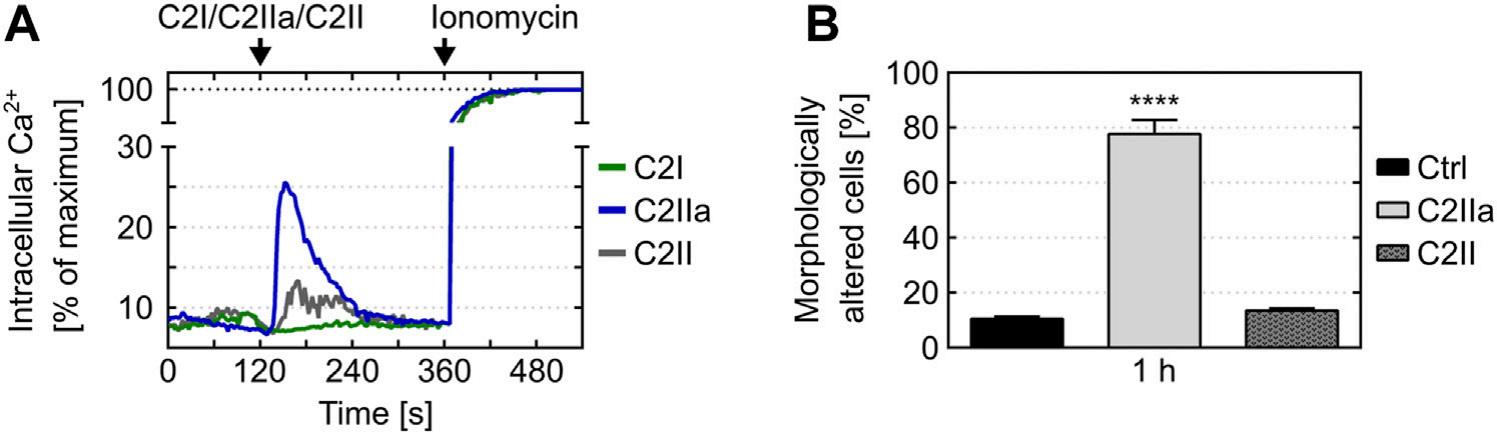
Effect of C2IIa, C2II and C2I on Ca^(2+)^ mobilization and morphological changes in primary human PMNs. **(A)** Treatment with C2IIa induces rapid Ca^2+^ influx into PMNs. Primary human PMNs were loaded with indo-1-AM. After pre-warming to 37°C, the cells were loaded into the flow cytometer and Ca^2+^ baseline was determined for 2 min. Afterwards, the cells were treated for 4 min with C2IIa (5 μg/ml), C2II (5 μg/ml) or C2I (1 μg/ml) and subsequently for 3 min with ionomycin. The individual points of the curves correspond to time in full seconds *versus* the medians of all fluorescence ratios representing [Ca^2+^]_i_, following their normalization according to the maximum values reached after addition of 2 μm ionomycin. Data is representative for three independent experiments. **(B)** Treatment with C2IIa but not with C2II induces changes in PMN morphology. Primary human PMNs were treated at 37°C with either C2IIa (5 μg/ml) or C2II (5 μg/ml) or without any protein as control (Ctrl). Pictures from the cells were taken after 1 h and quantitative analysis of morphological changes of PMNs was performed. Data show the results of 3 independent experiments, each with 3 technical replicates per condition. Values represent the mean ± SEM (n = 9). Significance was tested against the untreated control (Ctrl) using Kruskal–Wallis test with Dunn’s correction for multiple comparison (****p < 0.0001).

Cells were protected from C2IIa-induced morphological changes when they were incubated with C2IIa in the presence of established C2IIa pore blockers HA1568 (Bronnhuber et al., 2014) or quinacrine (Kronhardt et al., 2016) (Figure 2A), confirming that the observed effect was mediated by transmembrane pores formed by C2IIa. Pretreatment of cells with bafilomycin A1, which inhibits the acidification of endosomal vesicles and protects cells from intoxication with complete C2 toxin (Barth et al., 2000), had no effect on the C2IIa-induced changes in morphology (Figure 2B). Since acidification is crucial for pore formation by C2IIa in endosomes, this result implicates that the observed morphological changes after treatment with C2IIa are not induced by endosomal pore formation. To investigate the potential role of C2IIa pore formation in the plasma membrane of PMNs in more detail, the intracellular Ca^2+^ signal upon treating primary human PMN with C2IIa, its inactive precursor C2II and C2I were measured by flow cytometry. As shown in Figure 3A and Supplementary Figure S3, there was rapid increase of intracellular free Ca^2+^ concentration in PMNs after addition of C2IIa, while no comparable effect was measured after addition of C2II or C2I. This is in accordance with pore formation by C2IIa in the plasma membrane of PMNs, which seems to be crucial for the observed effects on PMNs, since morphological changes on PMNs can only be observed after treatment with C2IIa, but not with C2II (Figure 3B).

**FIGURE 4:**
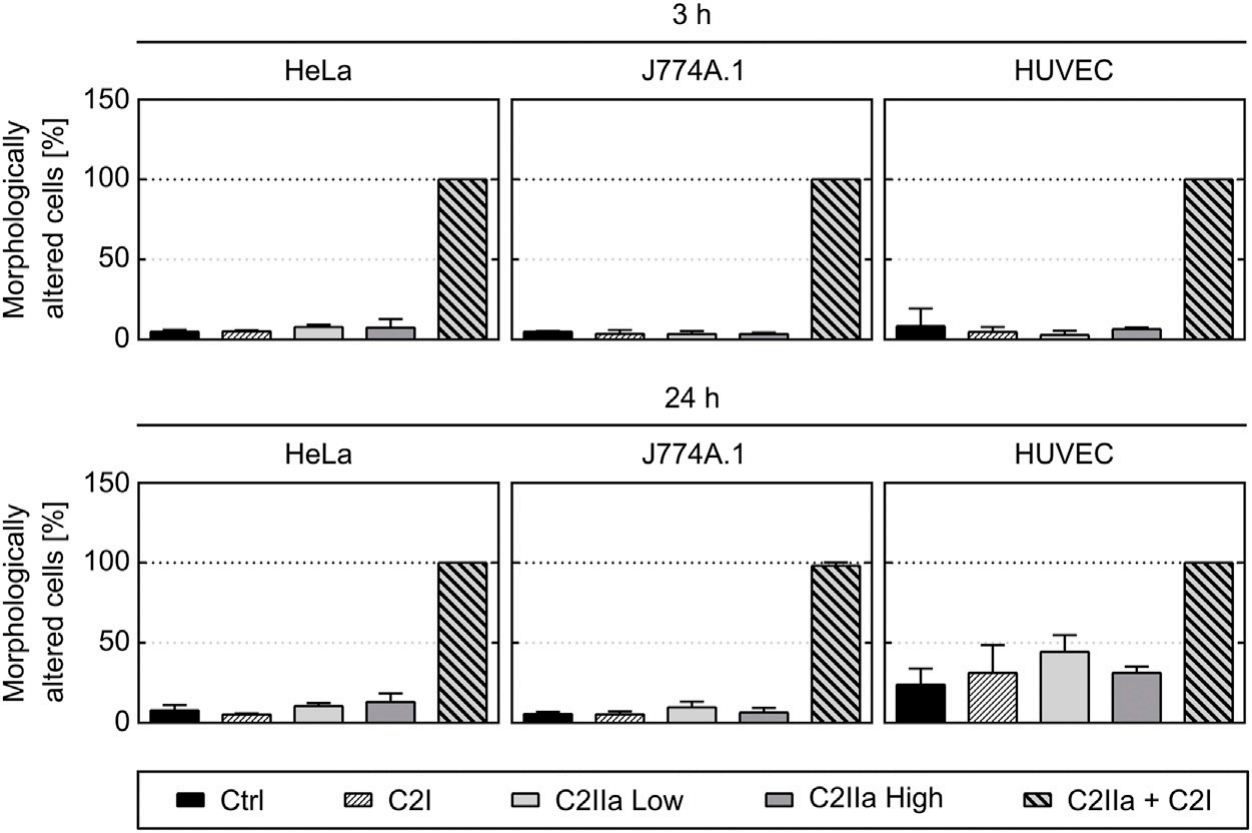
Effect of complete C2 toxin and its single components C2IIa and C2I on the morphology of HeLa, HUVEC and J774A.1 cells. The percentage of morphologically changed cells was quantified after 3 and 24 h treatment (37°C) of different cell types with C2I (1 μg/ml), C2IIa Low (2 μg/ml), C2IIa High (5 μg/ml), the binary toxin (2 μg/ml C2IIa + 1 μg/ml C2I) or without any protein as control (Ctrl). Data is representative for at least three independent experiments per time point. Values represent the mean ± SD of 3 technical replicates.

**FIGURE 5:**
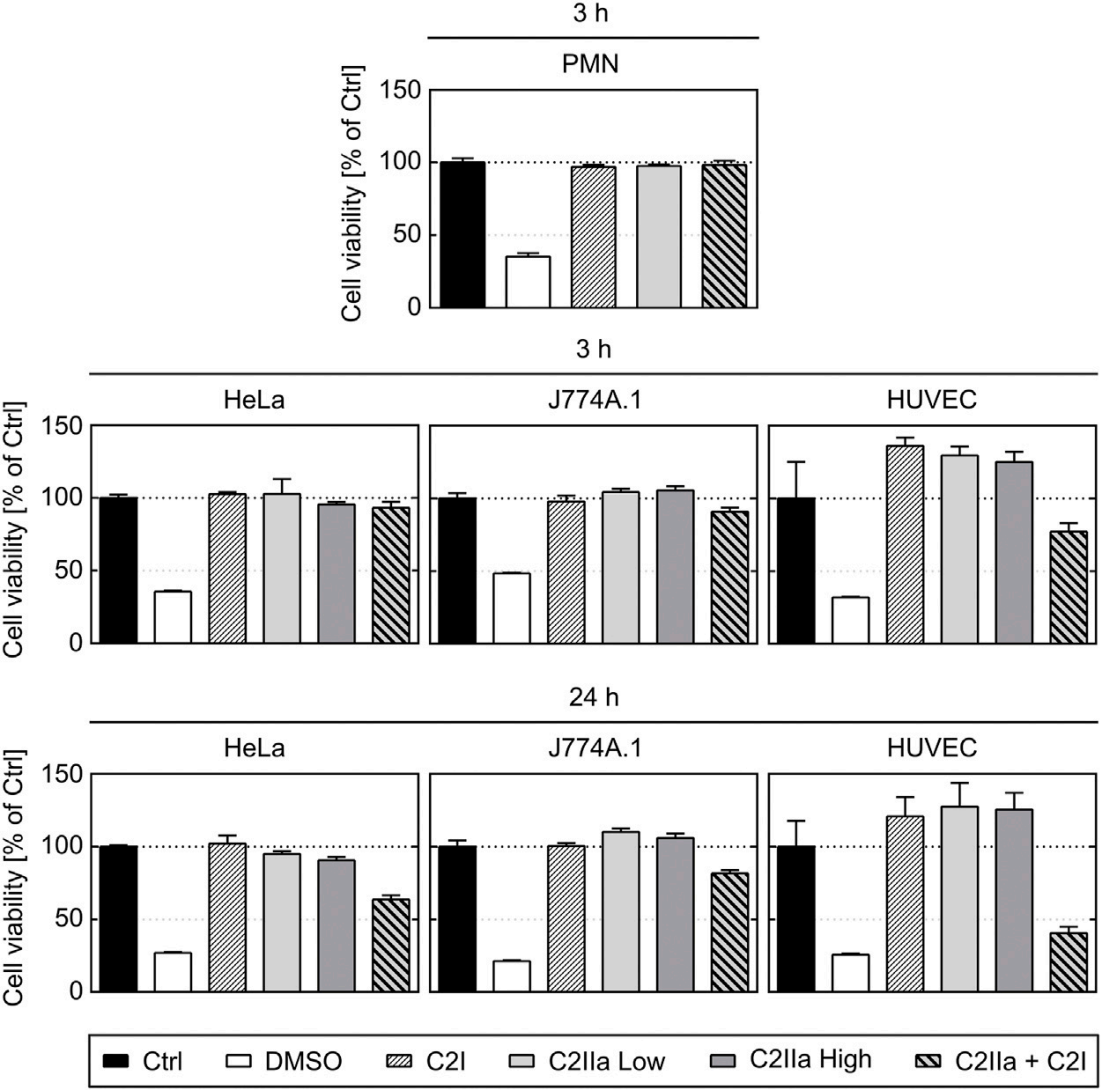
Effect of complete C2 toxin and its single components C2IIa and C2I on the viability of primary human PMNs and on HeLa, HUVEC and J774A.1 cells. Relative viability (% of control) of different cell types after 3 and 24 h incubation (37°C) with DMSO (20%), C2I (1 μg/ml), C2IIa Low (2 μg/ml), C2IIa High (5 μg/ml), the binary toxin (2 μg/ml C2IIa + 1 μg/ml C2I) or without any component as control (Ctrl) was measured by MTS assay. Data is representative for at least three independent experiments per time point. Values represent the mean ± SD of 3 technical replicates.

Prompted by the unexpected observation that C2IIa alone affects PMNs, we investigated the effect of C2IIa in direct comparison to C2IIa + C2I on other cell types including human epithelial cells (HeLa), human endothelial cells (HUVEC) and a murine macrophage line (J774A.1). Therefore, not only morphological changes, but also the viability of all cell types including PMNs were analyzed. As a control in the viability assays, DMSO was added to induce cell death. As shown in Figures 4, 5, all cell types responded to complete C2 toxin (i.e., C2IIa + C2I), but not to C2I or C2IIa alone, even not when C2IIa was applied in the culture medium for 24 h in a higher concentration (5 μg/ml) compared to that used for PMN-intoxication (2 μg/ml). Moreover, C2IIa + C2I, but not C2I or C2IIa alone, reduced the amount of viable cells after 24 h of incubation (Figure 5, lower panel). This was expected from earlier reports that C2 toxin induces apoptotic cell death after 24–48 h (Heine et al., 2008). After 3 h, all cell types including human primary PMNs were viable after treatment with C2IIa + C2I and C2IIa alone (Figure 5, upper and middle panel). Moreover, an incubation for 6 h with either C2IIa or complete C2 toxin (C2IIa + C2I) did not reduce the viability of primary human PMNs *ex vivo* (Supplementary Figure S4). Since primary PMNs do not survive for 24 h *ex vivo*, the effect of C2IIa on their viability could not be analyzed for prolonged incubation periods.

Taken together, the comparison between the different cell types confirmed earlier results that all cell types respond to the combination C2IIa + C2I, while treatment with the single components of C2 toxin had no effect on the tested cell types, except for PMNs, which selectively responded to C2IIa alone.

### C2IIa Reduces the Chemotactic Translocation of Primary Human PMNs

**FIGURE 6:**
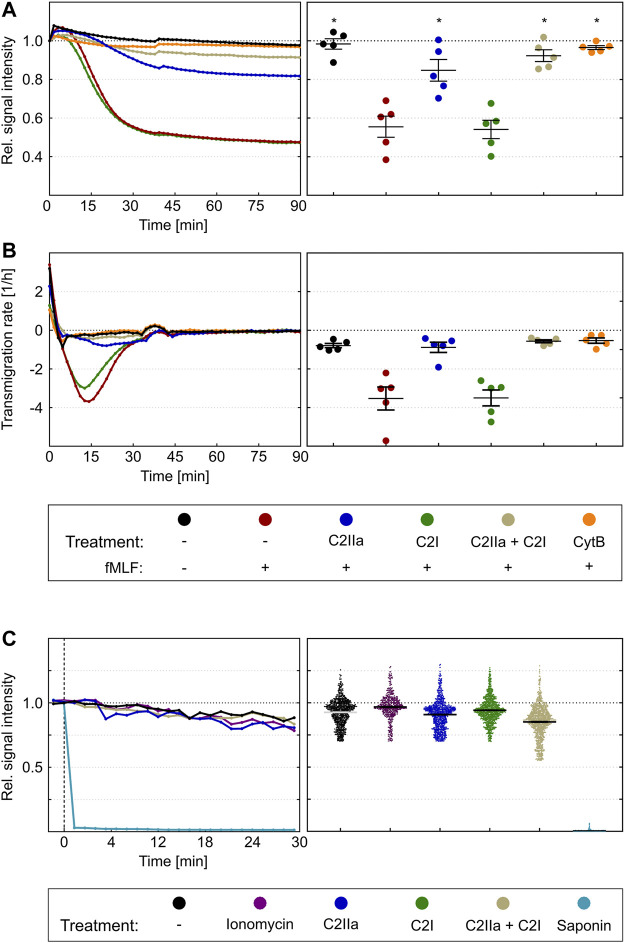
Effect of complete C2 toxin and its single components C2IIa and C2I on the chemotactic translocation of primary human PMNs *ex vivo*. **(A)** Calcein stained PMNs were applied to the upper site of cell-culture inserts with 3 µm pores in the presence or absence of C2IIa (2 μg/ml), C2I (1 μg/ml), the binary toxin (2 μg/ml C2IIa + 1 μg/ml C2I) or cytochalasin B (5 μg/ml) as a positive control. After an intoxication time of 2 h, fMLF (10 nM) was added to the lower compartment to trigger chemotaxis. Calcein fluorescence was measured from the top immediately after stimulation over a period of 90 min. The decline in signal intensity is considered to be a measure of the fraction of cells migrated from the upper to the lower compartment. Its computation is described in the methods section. The left panel shows one representative recording. The right panel summarizes results of five independent experiments after 90 min. Values are given as mean ± SEM (n = 5). Significance was evaluated with the Kruskal–Wallis test and Dunn’s correction for multiple comparison (*p < 0.05). Significant differences are all with respect to fMLF control samples. Symbols and colours are identical for **(A)** and **(B)**. **(B)** First time derivative of signals from experiments in **(A)** representing the transmigration rate [1/h]. The statistical analysis was performed with values of the maxima of the transmigration rates per individual experiment (n = 5), confirms an inhibition by the binary C2 toxin and the C2IIa component. **(C)** The left panel shows representative Fx (Fluorescence value at an timepoint x)/F0 curves of single cells over time. Immediately after addition of saponin the calcein signal is completely lost. On the opposite, treatment with ionomycin (2 µM), C2IIa (2 μg/ml), C2I (1 μg/ml) or both components (2 μg/ml C2IIa + 1 μg/ml C2I) show fluorescent signals similar to the untreated control. Right panel: Scatter plot shows F30/F0 for controls and treatment with ionomycin, C2IIa, C2I, both components of C2 toxin (C2IIa + C2I) or saponin. Values are given as mean ± SEM (Data are obtained from 3 independent experiments).

To investigate whether C2IIa also affects PMN functions, the chemotactic activity of primary human PMNs was analyzed in a dual chamber system. To this end, PMNs were stained with the fluorescence dye calcein and added to the upper side of cell-culture inserts with 3 µm pores. Pores allow cells to migrate from the top to the bottom compartment of the chamber along a chemotactic gradient (Davidson and Patel, 2014). The PMNs in the upper chamber were incubated for 2 h in the presence or absence of either C2IIa, C2I, C2 toxin (C2IIa + C2I) or cytochalasin B as an established migration-inhibiting compound. Subsequently, the chemoattractant fMLF was added to the lower compartment to trigger PMN chemotaxis. The amount of translocated PMNs was measured via the decline in the upper calcein fluorescence immediately after stimulation with fMLF over a period of 90 min. As expected, cytochalasin B and the binary C2 toxin (C2IIa + C2I) provoked a complete inhibition of fMLF-dependent translocation of PMNs, while C2I alone had no effect (Figure 6A). However, treatment with C2IIa alone resulted in a significant reduction of the amount of translocated PMNs, implicating that C2IIa inhibits the chemotactic migration of human PMNs *ex vivo*. This finding is further affirmed by the fact, that the fastest rate of PMN transmigration was drastically haltened by C2IIa but not C2I treatment (Figure 6B), suggesting that this mode of intoxication has the potential to effectively reduce leukocyte mass movement (Getter et al., 2019).

Since the described measurement is dependent on persistence of calcein in the cytosol of PMNs and C2IIa seem to be related to pore formation in the plasma membrane, a control experiment was performed to analyze whether treatment of PMNs with C2IIa leads to an efflux of calcein, a much larger molecule than Ca^2+^, from the cells. Potentially, this could then result in a decreased fluorescent signal of C2IIa-treated PMNs and false-positive results. Therefore, calcein-stained PMNs were treated with either saponin, which is known to disrupt cell membranes, with the pore formation inducer ionomycin, with C2IIa, C2I, the complete binary C2 toxin (C2IIa + C2I), or with bath solution as control. As expected, saponin-treatment resulted in a complete loss of the fluorescence signal, which was calculated as the ratio of fluorescence between timepoint 30 min (F30) and timepoint zero (F0) (Figure 6C). In contrast, F30/F0 signals of PMNs exposed to any of the other compounds did not change (Figure 6C). This result excludes the possibility that the observed reduction of calcein is not a sole indicator of cellular translocation but also due to calcein loss through C2IIa pores in the plasma membrane.

### C2IIa Reduces the Transendothelial Chemotactic Translocation of Primary Human PMNs But Does Not Affect the Endothelial Barrier Integrity

**FIGURE 7:**
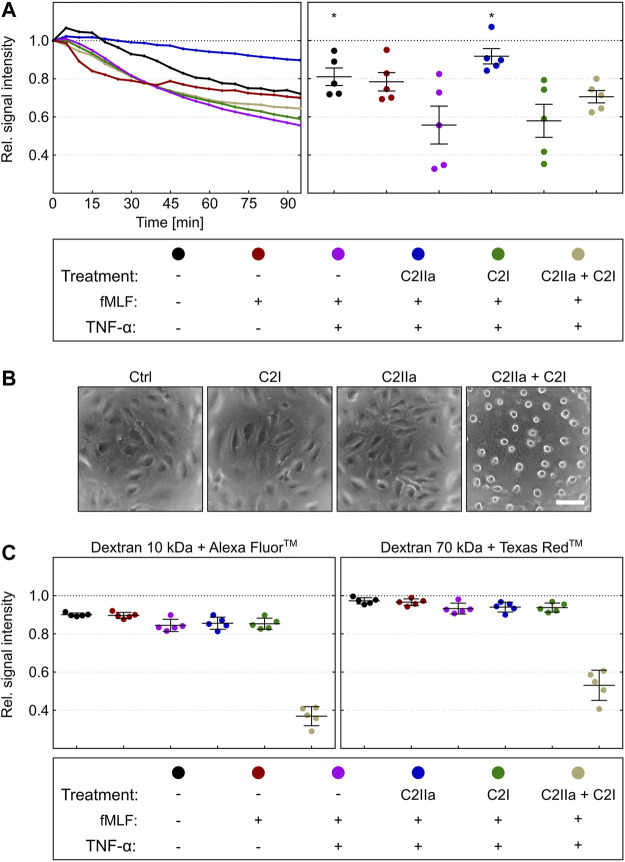
Effect of complete C2 toxin and its single components C2IIa and C2I on the transendothelial chemotactic translocation of primary human PMNs *ex vivo* and on barrier integrity. **(A)** An endothelial monolayer (HUVEC) was cultured on filter inserts with 3 µm pore size and activated by TNFα (10 ng/ml). Calcein-stained PMNs were pre-incubated in the presence or absence of C2IIa (2 μg/ml), C2I (1 μg/ml) or both components (2 μg/ml C2IIa + 1 μg/ml C2I) for 2 h and subsequently seeded on the apical site of endothelial cells. PMN chemotaxis towards the lower chamber was stimulated by addition of fMLF (10 nM). Calcein fluorescence was measured from the top immediately after stimulation over a period of 95 min. The decline in signal intensity is considered to be a measure of the fraction of cells migrated through the endothelial monolayer within 95 min. Its computation is described in the methods section. The left panel shows one representative recording. The right panel summarizes results of five independent experiments after 95 min. Values are given as mean ± SEM (n = 5). Significance was tested with Wilcoxon matched-pairs signed rank test (one-tailed) by GraphPad software (*p < 0.05). Statistical tests refer to samples treated with fMLF + TNFα. **(B)** Representative pictures of HUVEC cells in a monolayer which were treated for 3 h at 37°C with solvent (Ctrl), C2IIa (2 μg/ml), C2I (1 μg/ml) or both components (2 μg/ml C2IIa + 1 μg/ml C2I). **(C)** The treatment of the co-culture (HUVEC) with C2IIa does not increase transendothelial fluxes of fluorescence dye-labelled dextrans with molecular weights of either 10 kDa or 70 kDa (left and right panels, respectively). In contrast to the single C2 toxin components, the whole toxin (2 μg/ml C2IIa + 1 μg/ml C2I) disrupted the endothelial barrier-integrity. Values are given as mean ± SEM of five independent experiments (n = 5).

Prompted by this finding, we analyzed the chemotactic translocation of PMNs in co-culture with human endothelial cells, which is a better *in vitro* model of the physiological extravasation process (Filippi, 2016). To this end, a tight monolayer of human endothelial cells (HUVEC) was grown in the dual chamber device and activated by treatment with TNFα (Makó et al., 2010; Schildberger et al., 2010; Mehta and Malik, 2006). Calcein-stained PMNs were pre-incubated for 2 h in the presence or absence of either complete C2 toxin (C2IIa + C2I) or the single components C2IIa or C2I, respectively, and subsequently seeded on the apical side of activated endothelial monolayers. As described before, PMNs were stimulated for transmigration by application of fMLF into the lower chamber. The amount of translocated PMNs was detected by fluorescence measurement over 95 min (Figure 7A, left panel) and the results of five independent experiments were evaluated (Figure 7A). These findings revealed that C2IIa alone significantly reduced the transmigration. As expected, C2I had no detectable effect. However, HUVEC cells rounded up after treatment with complete C2 toxin, indicating that they are sensitive for C2 toxin and able to bind C2IIa under these experimental conditions. Importantly, C2IIa did not affect the integrity of the endothelial barrier formed by HUVEC cells (Figures 7B,C). Treatment of the HUVEC/PMN co-culture with C2IIa did not increase the transendothelial fluxes of fluorescence dye-labelled dextrans with molecular weights of either 10 or 70 kDa (Figure 7C, left and right panels, respectively). In contrast, complete C2 toxin (C2IIa + C2I) disrupted the endothelial barrier-integrity. In conclusion, the results suggest that C2IIa inhibits chemotactic diapedesis of human PMNs but does not exhibit cytotoxic effects on the endothelial cells.

## Discussion

Prompted by recent reports that the activated transport subunit of the binary C2 toxin, C2IIa, triggers the exocytosis of enzymes from lysosomes via Ca^2+^ influx through C2IIa pores formed in the plasma membrane of cells (Nagahama et al., 2017; Nagahama et al., 2021), we investigated the effect of C2IIa on various cell types. Taken all together, we found that C2IIa had no effects on cell morphology or viability of epithelial and endothelial cells as well as macrophages, but changed the morphology of primary human PMNs in a time- and concentration-dependent manner. However, concentrations of C2IIa in culture media used in the present experiments were higher than those required in combination with C2I for an effective intoxication of cells. The observed effect of C2IIa on the morphology of PMNs was prevented in the presence of compounds, which were previously described as blockers of C2IIa pores *in vitro* and in living cells (Bronnhuber et al., 2014; Knapp et al., 2016; Kronhardt et al., 2016; Kreidler et al., 2017), implicating that the effect of C2IIa on PMN morphology was mediated by C2IIa-dependent pore formation. Moreover, C2II, the precursor protein of C2IIa that does not form trans-membrane pores (Schmid et al., 1994) had no detectable effect on PMN morphology, supporting the hypothesis that C2IIa pores induce the changes in PMN morphology. However, bafilomycin A1, which prevents acidification of endosomal vesicles and protects cells from C2 toxin, had no effect on the C2IIa-induced changes of PMN morphology. This strongly suggests that C2IIa pores, which mediate the observed effect on PMNs, are formed in the cytoplasmic membranes of PMNs rather than in their endosomal membranes. This is supported by the finding that treatment of PMNs with C2IIa but not with C2II immediately increased the cytoplasmic amount of Ca^2+^, most likely via influx at the plasma membrane caused by C2IIa pores.

*In vitro*, C2IIa forms pores in artificial black lipid bilayer membranes also at neutral pH conditions (Schmid et al., 1994), but pore formation is increased under acidic conditions (Bachmeyer et al., 2001). This is plausible since C2IIa trans-membrane pores are formed in the membranes of acidified endosomes for the delivery of C2I from the endosomal lumen into the host cell cytosol (Barth et al., 2000; Haug et al., 2003a; Haug et al., 2003b; Kaiser et al., 2009; Nagahama et al., 2009; Ernst et al., 2017). By measuring the release of radioactively labelled rubidium from cultured living cells, we discovered earlier that C2IIa, but not the inactive precursor C2II, forms pores in plasma membranes, but this pore formation essentially required acidic conditions while no detectable pore formation was measured in cells under neutral pH conditions (Barth et al., 2000; Bachmeyer et al., 2001; Blöcker et al., 2003a), suggesting that the number of C2IIa pores that are formed in the plasma membranes of cells to initiate the C2 toxin uptake via Ca^2+^ influx is likely limited. This could also explain why treatment with C2IIa in the absence of C2I did not induce cytotoxic effects or obvious morphological changes in the variety of cell types tested so far, except of PMNs, where Ca^2+^ influx at the plasma membrane and altered calcium homeostasis integrates chemotactic and adhesive signals (Schaff et al., 2008). In conclusion, the results of this present study strongly suggest that in the case of C2IIa, the pore formation by this protein in the plasma membrane is the cause for reduction in chemotaxis of human PMNs *ex vivo*.

The transport subunits of the related clostridial binary actin ADP-ribosylating iota toxin of *C. perfringens* and CDT of *Clostridioides difficile* (Popoff et al., 1988; Perelle et al., 1997a), Ib and CDTb, respectively, exhibit cytotoxic effects in the absence of their enzyme subunits by pore formation in the plasma membranes of mammalian cells even under neutral conditions (Nagahama et al., 2011; Landenberger et al., 2021; Ernst et al., 2021)). Incubation of cells with Ib (Nagahama et al., 2014) or CDTb (Ernst et al., 2021; Landenberger et al., 2021) results in morphological changes and loss of viability. However, cells are protected by pore blockers (Ernst et al., 2021; Landenberger et al., 2021), confirming that the cytotoxic effects of Ib or CDTb in the absence of their enzyme subunits is mediated by pore formation. C2IIa shows structure and function homology to Ib and CDTb (Perelle et al., 1997b; Stiles et al., 2014; Blonder et al., 2005) but the toxins show some differences in their cellular uptake mechanisms regarding the endocytotic pathways and the mechanisms underlying translocation of enzyme subunits from endosomes into the cytosol (Blöcker et al., 2001; Hale et al., 2004; Gibert et al., 2007; Gibert et al., 2011). Moreover, while C2IIa binds via carbohydrate structures to all cell types (Eckhardt et al., 2000), Ib and CDTb both exploit the surface protein LSR as a receptor (Papatheodorou et al., 2011). Therefore, only LSR-expressing cells respond to the toxins iota or CDT and to their transport subunits Ib or CDTb, respectively (Papatheodorou et al., 2011). In conclusion, the ability of C2IIa to exhibit cytotoxicity only to a specific cell type although it can bind to all cell types is unique among these three toxins.

Our results revealed that treatment with C2IIa reduced the chemotactic translocation of primary human PMNs *ex vivo* through a porous membrane and across the barrier of human endothelial cells. We could exclude that a loss of the calcein dye from the cells contributes to the detected decrease of fluorescence in these experiments with calcein-stained PMNs. Noteworthy, C2IIa did not affect endothelial cells or the barrier integrity of the endothelial monolayer, confirming the PMN-selectivity of C2IIa. Therefore, the findings suggest that C2IIa alone selectively targets PMNs and down-modulates their chemotactic translocation. This mode of action towards these important cells of the innate immune system might represent a pathophysiological contribution to the already established mode of action of the complete binary C2 toxin. Moreover, these novel findings might pave the way towards pharmacological strategies for targeted down-modulation of the excessive and detrimental chemotactic PMN translocation, which was observed in the context of (post-)traumatic hyper-inflammatory conditions, for example, after blunt chest trauma and the associated lung contusion (Perl et al., 2012). Here, the local application of C2IIa could be beneficial to reduce the amount of PMNs extravasating into lung parenchyma without affecting other pulmonary cell types such as alveolar epithelial cells or macrophages.

## Data Availability

The raw data supporting the conclusions of this article will be made available by the authors, without undue reservation.

## Supplementary Material

Supplementary Figure S1 Analysis of recombinant C2I, C2II and C2IIa. The recombinant proteins were purified and C2IIa was activated as described in *Materials and Methods*. Proteins were subjected to 12.5% SDS-PAGE and analyzed by Coomassie blue staining (750 ng of each protein, left panel) or by Western blotting with specific antisera against C2I or C2II (350 ng of each protein, right and middle panel, respectively).

Supplementary Figure S2 Effect of C2IIa on the morphology of primary human PMNs. Primary human PMNs were incubated without (Ctrl.) or with increasing concentrations of C2IIa at 37°C and analyzed by phase contrast microscopy. Pictures of the cells were taken 2 min before (indicated as untreated, UT) and immediately after protein addition into the medium (indicated as time point 0) and after 0.5, 1 and 6 h. The percentage of morphologically changed cells was quantified from the pictures. Data is representative for three independent experiments. Values represent the mean ± SD of three technical replicates.

Supplementary Figure S3 The ability of C2IIa to mediate intracellular Ca^2+^ mobilization depends on the level of C2IIa stimulation. Primary human PMNs were loaded with Indo-1-AM. After pre-warming to 37°C, the cells were applied to the flow cytometer and the Ca^2+^ baseline was determined for 2 min. Subsequently, the cells were treated for 4 min with 0.2, 0.6, 1, 2, 5 and 10 µg/ml of C2IIa or with the PBS as control. Subsequently, ionomycin was added to the samples for 3 min. The individual points shown in the curves correspond to the times in full seconds versus the medians of all fluorescence ratios representing [Ca^2+^]_i_, following their normalization according to the maximal values reached after addition of 2 μM ionomycin. Data is representative for three independent experiments.

Supplementary Figure S4 Effect of C2IIa and C2IIa + C2I on the viability of primary human PMNs *ex vivo*. Primary human PMNs were incubated without any protein (Ctrl.) or with either C2IIa (2 µg/ml) or complete C2 toxin (2 µg/ml C2IIa + 1 µg/ml C2I) at 37°C. After 6 h, the amount of viable cells was determined with the MTS cell viability assay and the percentage of viable cells calculated. Data is representative for three independent experiments. Values represent the mean ± SD of three technical replicates.
