# Supplementary figures and images for: The Pore-Forming Subunit C2IIa of the Binary Clostridium botulinum C2 Toxin Reduces the Chemotactic Translocation of Human Polymorphonuclear Leukocytes
Source: Front Pharmacol. 2022 Feb 11;13:810611. doi: 10.3389/fphar.2022.810611 (PMC8881014; doi:10.3389/fphar.2022.810611)

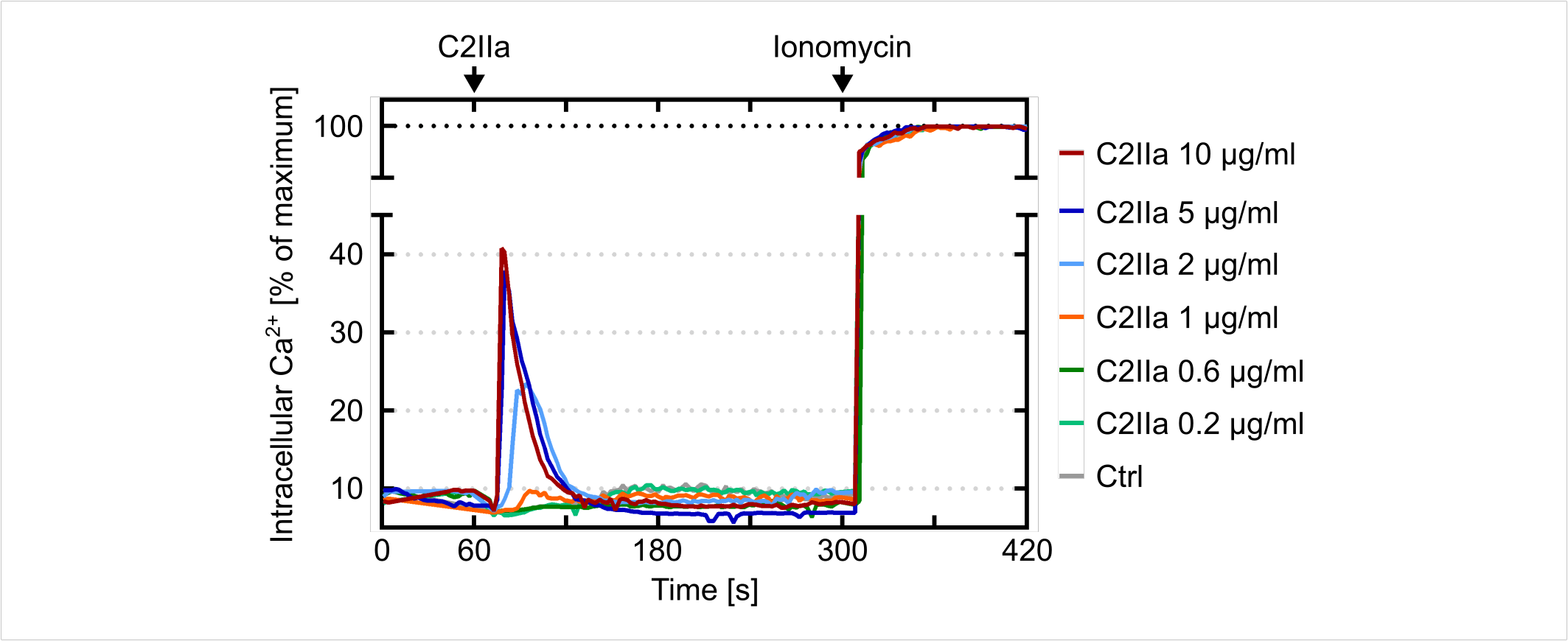

Supplement: Supplementary file 1 [file Image3.TIF]

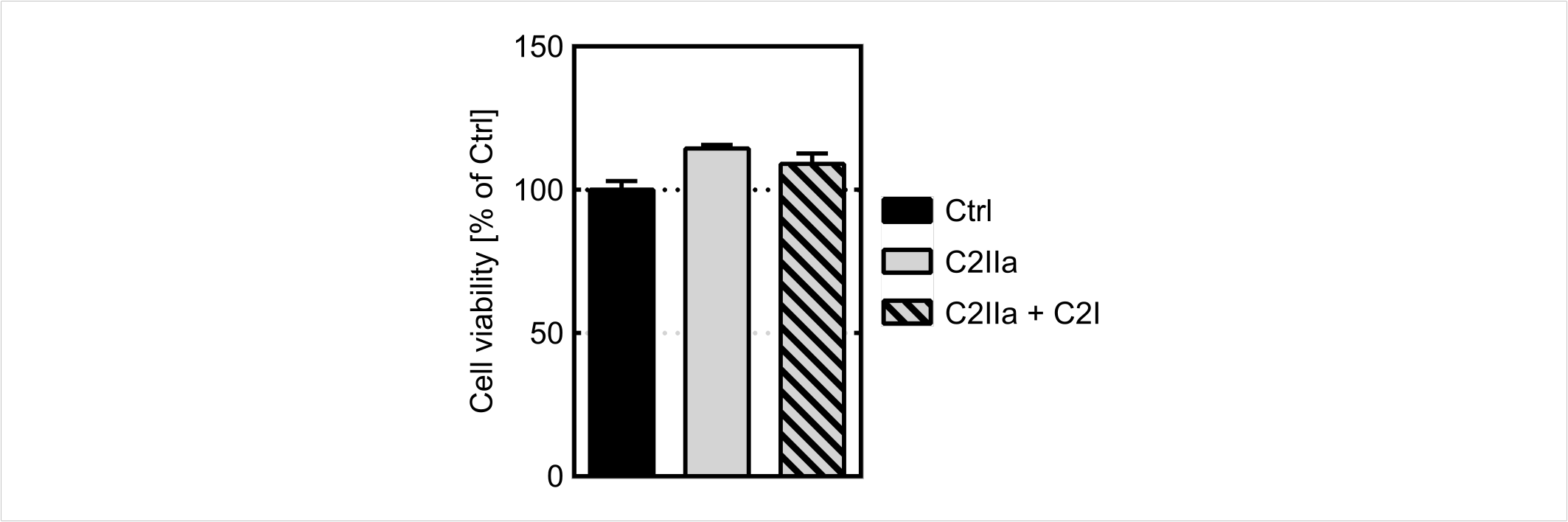

Supplement: Supplementary file 2 [file Image4.TIF]

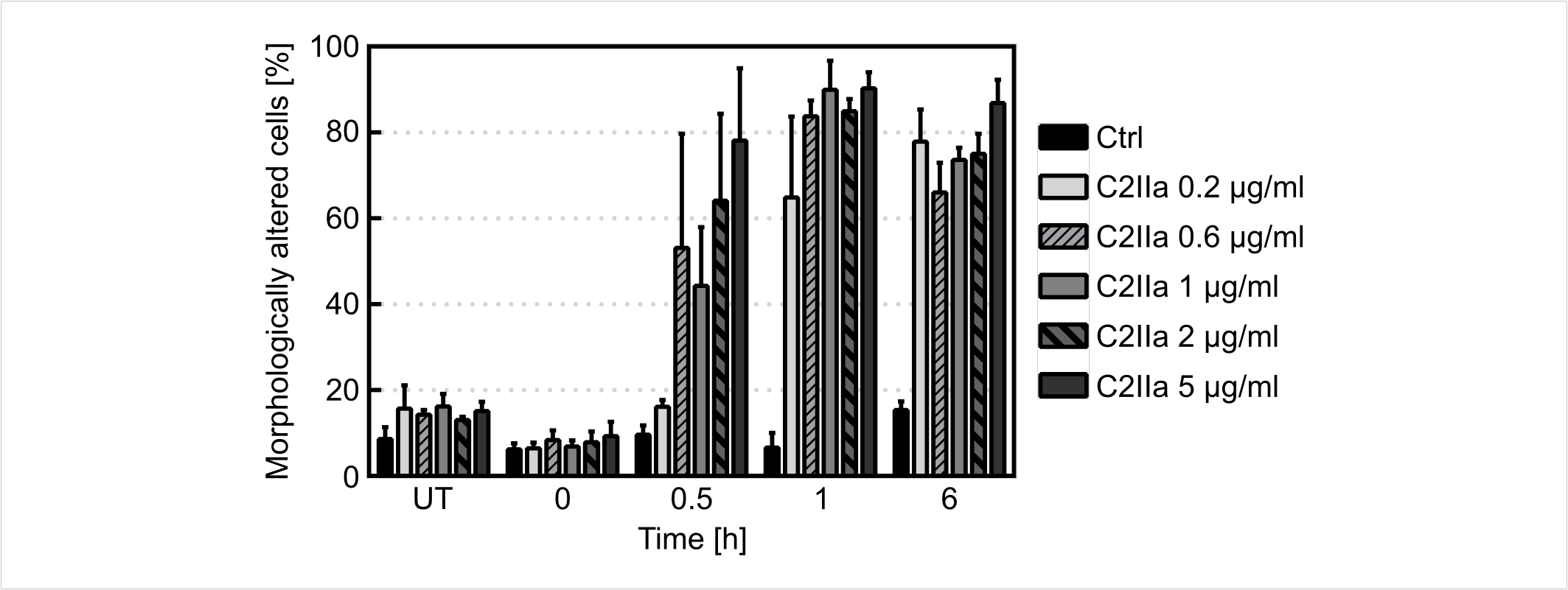

Supplement: Supplementary file 3 [file Image2.TIF]

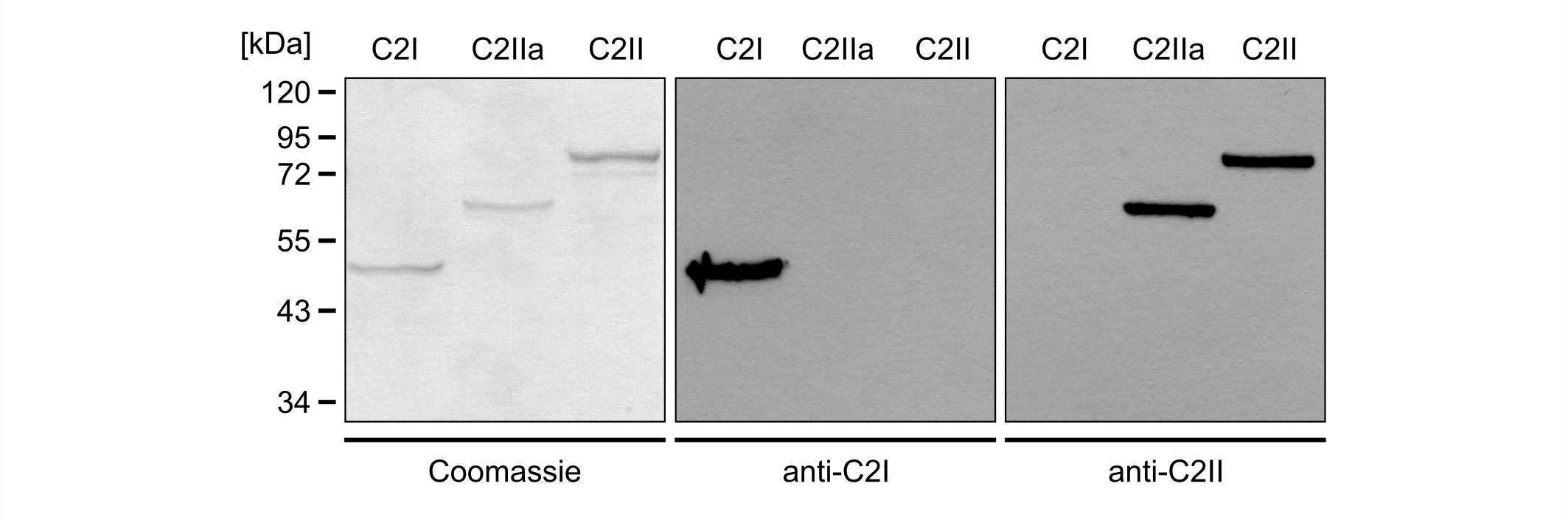

Supplement: Supplementary file 4 [file Image1.TIF]
